# Improving the efficiency of breast radiotherapy treatment planning using a semi‐automated approach

**DOI:** 10.1002/acm2.12006

**Published:** 2016-11-30

**Authors:** Robert A Mitchell, Philip Wai, Ruth Colgan, Anna M Kirby, Ellen M Donovan

**Affiliations:** ^1^ Joint Department of Physics The Royal Marsden NHS Foundation Trust/Institute of Cancer Research Sutton Surrey UK; ^2^ Department of Radiotherapy The Royal Marsden NHS Foundation Trust Sutton Surrey UK

**Keywords:** automated planning, autosegmentation, breast, dosimetry, radiotherapy

## Abstract

**Objectives:**

To reduce treatment planning times while maintaining plan quality through the introduction of semi‐automated planning techniques for breast radiotherapy.

**Methods:**

Automatic critical structure delineation was examined using the Smart Probabilistic Image Contouring Engine (SPICE) commercial autosegmentation software (Philips Radiation Oncology Systems, Fitchburg, WI) for a cohort of ten patients. Semiautomated planning was investigated by employing scripting in the treatment planning system to automate segment creation for breast step‐and‐shoot planning and create objectives for segment weight optimization; considerations were made for three different multileaf collimator (MLC) configurations. Forty patients were retrospectively planned using the script and a planning time comparison performed.

**Results:**

The SPICE heart and lung outlines agreed closely with clinician‐defined outlines (median Dice Similarity Coefficient > 0.9); median difference in mean heart dose was 0.0 cGy (range −10.8 to 5.4 cGy). Scripted treatment plans demonstrated equivalence with their clinical counterparts. No statistically significant differences were found for target parameters. Minimal ipsilateral lung dose increases were also observed. Statistically significant (*P* < 0.01) time reductions were achievable for MLCi and Agility MLC (Elekta Ltd, Crawley, UK) plans (median 4.9 and 5.9 min, respectively).

**Conclusions:**

The use of commercial autosegmentation software enables breast plan adjustment based on doses to organs at risk. Semi‐automated techniques for breast radiotherapy planning offer modest reductions in planning times. However, in the context of a typical department's breast radiotherapy workload, minor savings per plan translate into greater efficiencies overall.

## 1 Introduction

Radiotherapy following breast conservation surgery improves local control and survival.^1^ There were 50,285 new cases of breast cancer diagnosed in the UK in 2011, approximately 50% of whom required radiotherapy.^2^, ^3^ Indeed breast radiotherapy makes up around 30% of most UK departments’ workloads.^4^ In this group of patients therefore, small reductions in the time required for any aspect of the radiotherapy planning and treatment pathways will result in large time and resource savings at the departmental level.

While the use of simple Intensity Modulated Radiation Therapy (IMRT) planning techniques improves dose homogeneity within the breast tissue,^5^, ^6^ they are currently more labor intensive, requiring greater planning expertise and longer planning times than wedge only compensation.^7^ In addition to this, the development of more complex treatment techniques for breast cancer patients, such as those incorporating simultaneous integrated boosts or internal mammary lymph node irradiation,^8^ will lead to a significant workload increase in terms of critical structure delineation. The development of outlining and planning automation techniques should therefore be considered essential for contemporary radiotherapy departments.

Strategies investigated to automate aspects of the breast planning process range from simple programming in the treatment planning system coupled with segment weight optimization,^9^, ^10^ to complete inverse IMRT.^11^ Hybrid IMRT approaches incorporate highly weighted open fields with fully inverse IMRT‐derived fields.^11^, ^12^ The hybrid approach described by Purdie et al. has been incorporated into the rayAutoBreast module of the RayStation treatment planning system (RaySearch Laboratories AB, Stockholm, Sweden).^13^ This technique will also automate the optimization of the whole breast treatment fields by means of a clinical decision hierarchy. Purdie et al. report having treated 1661 patients with plans generated using this method.

The objective of this study was to investigate the introduction of automation techniques into breast radiotherapy planning practice through commercially available autosegmentation software and noncomplex, noncommercial scripting solutions using the Pinnacle^3^ treatment planning system (Philips Radiation Oncology Systems, Fitchburg, WI, USA).

## 2 Methods

### 2.A Patient/plan data

The treatment plans for 40 clinical breast radiotherapy patients were selected consecutively from a reverse chronologically ordered list. All patients had consented at time of treatment for their images to be used for research purposes. Patients had previously been scanned using either a Philips Brilliance Big Bore (60‐cm field of view, 2‐mm slice thickness) or GE Lightspeed (50‐cm field of view, 1.25‐mm slice thickness) CT scanner. Outlining and planning were carried out on a Pinnacle^3^ v9.8 treatment planning system. Scripting was performed using the planning system's inbuilt programming language, which incorporates object‐oriented aspects. All patients were treated to a prescription dose of 40 Gy in 15 fractions with field energies of predominantly 6 MV; 10 MV was also used when necessary for patients with large chest wall separation. The median (range) of breast volumes treated was 952 cc (223–2697 cc). Of these, 18 were left‐sided treatments and 22 were right‐sided. Eight patients received irradiation to the supraclavicular fossa.

### 2.B Spice

The autosegmentation software used was the Smart Probabilistic Image Contouring Engine (SPICE), a purchasable module for Pinnacle^3^.^14^ The autosegmentation process applies rigid and deformable registrations together with probability‐based structure refinements. The modified atlas contours are subsequently added to the structure set. The suitability of the SPICE heart and lungs volumes for clinical use was investigated using a cohort of ten patients from the UK HeartSpare study.^15^ The volumes created were compared quantitatively to those defined by an experienced radiation oncologist using the Dice Similarity Coefficient (DSC), which is twice the ratio of the volume of the overlapping region to the sum of the two volumes. A comparison between mean heart doses was also performed.^14^

### 2.C Scripting

**Figure 1:**
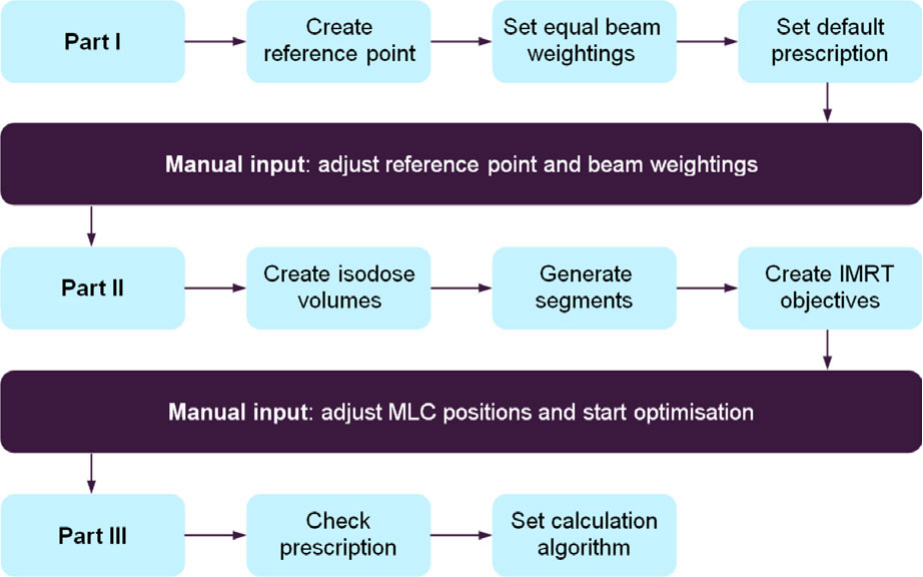
Simplified scripting workflow. Manual planner interaction is required following each part of the script. The different Linac MLC types are accounted for in Part II.

A script was developed (PW, RAM) with the aim of automating parts of the breast planning process in order to facilitate reductions in step‐and‐shoot planning times while maintaining plan quality. The script workflow is given in (Fig. 1); it was not the purpose of this script to automate tangential field placement. The first part of the script initially created the prescription point at the isocenter of the breast fields and set a default prescription of 40 Gy in 15 fractions; the beam weightings were also set for each beam to give equal contributions to the dose at the prescription point. The planner had then to derive the necessary minimum, uncompensated, dose coverage of the whole breast by renormalizing the open tangential field distribution to achieve 95% dose coverage.

The second part of the script detected the plan maximum dose and created a set of prespecified isodose volumes from 105% of the prescription dose up to approximately the maximum dose. The purpose of this was to introduce control points with Multileaf Collimator (MLC) shielding for dose distribution homogenization. For control point MLC leaves to conform to the isodose volumes, the script was required to first expand the isodose volumes beyond the anterior field edges. By utilizing the expanded isodose volumes as block structures within the treatment planning system's block creation tool, the script added control points with the MLC leaf positions required to homogenize dose at the specified isodose levels. Approximately the same number of control points were added for each field. To achieve good conformation, the isodose volume expansion direction was defined to be approximately parallel to the direction of MLC leaf travel. This was scripted by introducing gantry and collimator angle dependences into the expansion definitions; expansion magnitudes and directions were defined explicitly for set collimator and gantry angle ranges. The result was a step‐wise expansion of the isodose volumes, which consisted of several large anterior expansions coupled with smaller superior/inferior expansions/contractions.

Cardiac and lung MLC shielding were defined by the local Virtual Simulation clinical protocol implementation process. The Pinnacle^3^ block tool removed this from the shielding control points. The script restored the cardiac and lung shielding using the first control point on each field, which was an unmodified open‐field segment, identifying the posterior leaf bank using treatment field gantry and collimator angles, and then extracting the corresponding MLC leaf positions. These were applied to the remaining control points. From (Fig. 1), this was performed during the segment creation process. The script's functionality was not affected by the presence of additional fields, for example, to treat nodes in the supraclavicular fossa.

Control point Monitor Units (MU) were then derived using segment weight optimization. Pinnacle^3^ utilizes a gradient descent method during optimization; this particular technique optimizes the MU per segment in order to satisfy the dosimetric objectives.^16^ The optimization objectives added by the second part of the script were: uniform dose at the prescription dose (weighting of 90) and minimum dose at 95% of the prescription dose (weighting of 20). The weightings specified are simple multiplication factors applied to the individual objective values and only scale the contribution of each objective to the overall composite objective value. The script‐generated optimization volume, PTV_opt_, was defined by the original 95% isodose surface for the open fields and was found to be sufficient for the optimization process; this volume was generated alongside the other isodose volumes at the beginning of the second stage of the scripting workflow.

Scripting accounted for Linacs (Elekta Ltd, Crawley, UK) fitted with three different MLC types (MLC leaf width, maximum field size X by Y where X is the MLC defining direction): MLCi (10 mm, 40 cm by 40 cm), Agility (5 mm, 40 cm by 40 cm), and Beam Modulator (4 mm, 16 cm by 21 cm). Furthermore, the Beam Modulator MLC has fixed jaws, MLCi has movable X and Y jaws and Agility only has movable Y jaws according to IEC 61217 convention. The script was initially created for MLCi only but its applicability was subsequently extended to encompass all three MLC types.

### 2.D Planning

All planning was conducted by a single planner. The SPICE module was applied and plans were then created retrospectively using the scripts. All patients were further retrospectively planned manually and planning times were measured using a stopwatch. Dose calculations were performed using a collapsed cone convolution algorithm on a 0.25 × 0.25 × 0.25 cm^3^ resolution dose grid. Plan statistics were extracted using the whole breast, field‐based definition as per the IMPORT HIGH trial for the original and scripted plans.^17^ The whole breast volume was created from the 50% dose level, contracted by 10 mm superior/inferior and 5 mm posterior. This volume excludes the lungs expanded by 5 mm and the skin, which is taken to be 5 mm from the external contour. Dose homogeneity was quantified by the homogeneity index, defined as the ratio of the difference between the breast D_2%_ and D_98%_ to the D_50%_.^18^ Tests for normality were undertaken by histogram and Q‐Q plot inspection and the Wilcoxon Signed Rank Test was used to identify statistically significant differences between plan types.

The definition of planning time used in this study encompasses the derivation and subsequent MLC compensation of the dose distribution for the open treatment fields; it does not include the time taken for initial field placement. A difference in mean time of 5 min was considered an appropriate minimum threshold for the routine introduction of the automated method as this would equate to 1 h per working week if 12 breast cases were planned per week. A total of 35 cases were planned for MLCi and Agility treatment units which gave 90% power to detect a difference between manual and automated planning times at a significance level of 0.01.

## 3 Results

### 3.A Spice

A summary of the comparison between the heart and lungs volumes created by the clinician and those added by the SPICE thorax atlas is given in (Table 1). SPICE creates two heart volumes (Heart 1 and Heart 2); according to Bzdusek et al., this was found to be necessary during the acquisition of the ground truth patient data that constitute the atlas.^14^ More superior slices were outlined by the Heart 1 structure; however, the remaining slices corresponded exactly to those delineated by Heart 2. Heart 1 showed greater agreement with the clinician‐outlined heart with a mean DSC of 0.92 compared to 0.82 for Heart 2. Furthermore, the median difference in mean heart dose was smaller for Heart 1 than Heart 2 (0.0 cGy and 2.4 cGy, respectively) when compared to the clinician‐delineated heart. However, it should be noted that, owing to the limitations of out‐of‐field dose calculations, these statistics should be interpreted as estimates only.^19^

**Table 1:** Comparison between structures defined by an experienced clinician and those created by SPICE

Volumes	Heart 1	Heart 2	Lung–L	Lung–R
Clinician volume (cc)	586.2 (435.4–779.9)		2086.7 (1597.6–2548.5)	2394.6 (1842.4–3094.9)
SPICE volume (cc)	628.2 (501.5–816.6)	491.7 (370.8–666.0)	1960.5 (1471.1–2419.6)	2261.6 (1711.3–2965.3)
Overlap volume (cc)	562.0 (419.4–749.1)	442.8 (332.1–606.9)	1954.2 (1467.2–2411.4)	2253.7 (1700.3–2945.0)
DSC	0.92 (0.90–0.94)	0.82 (0.78–0.87)	0.97 (0.96–0.97)	0.97 (0.96–0.97)

Doses	Heart 1	Heart 2
Clinician mean dose (cGy)	73.4 (50.5–133.6)	
SPICE mean dose (cGy)	71.5 (51.0–133.1)	75.5 (50.5–143.0)
Difference (cGy)	0.0 (−10.8–5.4)	2.4 (−3.8–15.3)

Values are specified as median (range). The two SPICE‐generated heart structures were compared to a single, clinician‐defined heart for each patient.

### 3.B Planning

**Figure 2:**
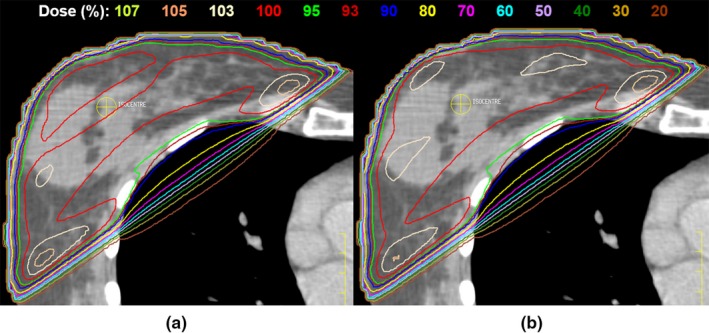
Representative axial dose distribution comparison between (a) clinical and (b) scripted plans. Isodoses are displayed as a percentage of the prescription dose.

Automated treatment plan quality was comparable to the original clinical plans in terms of dose homogeneity. No clinically relevant statistically significant differences were observed (*P* > 0.01) for the target parameters tested (Table 2). The dose distributions given in (Fig. 2) were representative for the patient cohort.

**Table 2:** Plan comparison between original clinical breast plans and scripted partial automation plans

	Original plan	Scripted plan	Difference	*P* value
**Plan parameters**				
MU^a^	309.3 (291.3–330.8)	308.9 (291.9–333.1)	−0.1 (−2.3–5.3)	0.83
Number of control points^a^	6.5 (5.0–10.0)	6.5 (5.0–10.0)	0.0 (−3.0–3.0)	0.19
**Breast**				
Mean dose (Gy)	40.2 (39.4–40.8)	40.3 (39.4–40.8)	0.0 (−0.4–0.4)	0.24
Maximum dose (Gy)	42.4 (42.0–43.4)	42.6 (41.9–43.2)	0.1 (−0.3–0.7)	0.02
Homogeneity index	0.12 (0.08–0.28)	0.11 (0.09–0.28)	0.00 (−0.01–0.01)	0.06
V_95%_ (%)	94.8 (86.5–99.1)	95.4 (88.1–99.1)	0.1 (−2.1–1.7)	0.11
Proportion of 103% (%)	11.3 (2.7–48.1)	11.8 (5.2–28.9)	1.4 (−27.6–12.2)	0.94
Proportion of 105% (%)	0.2 (0.0–4.3)	0.6 (0.0–4.9)	0.1 (−2.5–3.9)	0.06
Proportion of 107% (%)	0.0 (0.0–0.7)	0.0 (0.0–0.4)	0.0 (−0.3–0.0)	
**Organs at risk**				
Ipsilateral lung				
Mean dose (Gy)	4.5 (1.7–9.7)	4.6 (1.7–9.7)	0.0 (0.0–0.1)	< 0.01
V_20Gy_ (%)	7.8 (1.7–23.7)	7.8 (1.7–23.7)	0.0 (0.0–0.1)	< 0.01
Contralateral lung				
Mean dose (Gy)	0.2 (0.1–0.4)	0.2 (0.1–0.4)	0.0 (0.0–0.0)	0.22
Heart				
Mean dose (Gy)	0.8 (0.3–2.4)	0.8 (0.3–2.5)	0.0 (0.0–0.0)	0.04
V_5%_ (%)	5.9 (0.0–29.6)	5.9 (0.0–29.3)	0.0 (−0.3–0.2)	0.51
V_25%_ (%)	0.0 (0.0–3.1)	0.0 (0.0–3.1)	0.0 (0.0–0.0)	

a Tangential fields only.

Values quoted are median (range). The level for statistical significance is *P* < 0.01. The SPICE Heart 1 structure is used for reporting.

The planning times achieved for the different MLC types following manual retrospective planning are given in (Table 3). For MLCi and Agility, utilizing the automated script reduced planning times by 45% and 36% of the respective manual planning times (*P* < 0.01). Reductions in maximum planning times were also observed (16.1 min for MLCi).

**Table 3:** Planning time dependence on MLC type and planning method

MLC type	Manual time (min)	Scripted time (min)	Difference (min)	*P* value
MLCi	10.8 (7.5–36.3)	5.9 (4.9–20.2)	−4.9 (−16.1 to−2.7)	< 0.01
Agility	16.5 (11.7–21.3)	10.6 (7.8–16.3)	−5.9 (−8.6 to −1.3)	< 0.01
Beam Modulator	10.0 (6.9–16.1)	8.3 (6.2–13.5)	−0.7 (−3.1–0.0)	

Manual planning times were extracted from manual retrospective step‐and‐shoot plans. Values quoted are median (range). Note that planning times do not include initial field placement.

## 4 Discussion

This work investigated two aspects of automation: (I) an autosegmentation module and (II) use of the scripting feature in Pinnacle^3^. The aim was to reduce outlining and planning times while maintaining plan quality.

We have shown that SPICE gives clinically acceptable outlines for the heart and lungs (mean DSCs 0.92 and 0.97, respectively) and that the simple scripting solution allows for breast plans to be produced in shorter times whilst maintaining plan quality (6 min vs. 11 min for manual planning for MLCi). The planning time reductions offered by the script are modest (median 5 min MLCi, 6 min Agility, 1 min Beam Modulator) in a per plan context. However, the time saved in terms of the yearly workload of a typical department will be appreciable. For example, if a department plans 25 patients per week using the script and saves 5 min per patient, a time saving of approximately 14 working days could be achieved per year. If this technique was extended in the UK to approximately 25 Pinnacle^3^ centers, this would correspond to greater than a working year being saved across the UK.

Although the use of the breast script to aid planning does not completely eliminate the need for a final plan quality review by a physicist or dosimetrist, it does however, minimize the probability of planner‐induced errors. For larger patients and those with more complex and nonstandard shapes, the script may only be capable of providing a starting point for step‐and‐shoot in terms of the segment shapes. Nevertheless, the script will give the initial segment shapes quickly and the segment weight optimization will produce the best possible dose distribution to satisfy the specified optimization objectives, giving the planner more time to devise more sophisticated modifications to the existing segments in order to produce a clinically acceptable dose distribution. Subtle clinical differences between patients will always be challenging for treatment planning automation techniques.

Vicini et al. reported a median IMRT planning time of 45 min, in spite of using scripting to generate the MLC segments;^10^ note that this does not include the time required for field placement. The reduction in planning times in the current study may be partially attributed to advances in computational speed. Furthermore, the scripts developed by the authors have wider applicability than those described by Vicini et al. given their ability to cope with additional fields and prescriptions. A potential limitation of the Purdie study is that the breast volumes appear smaller (mean 663.6 ± 387.2 cc) than those in this study (median 952.1 cc).^12^ This may persist into the extended 1661 patient study, as the maximum patient separation is quoted as 21.6 ± 3.2 cm.^13^ However, their definition of patient separation is unspecified so a comparison is not possible. Nevertheless, since smaller breast volumes are often easier to plan, it would be interesting to investigate how their technique performs when planning larger and more complex breast volumes.

The mean DSCs obtained for the thorax structures are consistent with those given in the study by Zhu et al.; they report mean DSCs of 0.95 for the right and left lungs and 0.90 for the heart while the respective values in the current study are 0.97 and 0.92.^20^ Furthermore, given the small difference in mean heart dose between the SPICE‐defined Heart 1 structure and the clinician‐delineated heart, the Heart 1 volume was deemed to be suitable for use with all clinical breast radiotherapy patients. This brings breast radiotherapy planning practice into concordance with the majority of other tumor sites. In the context of the planning study, the lack of statistically significant changes in mean dose to the heart for the original and scripted plans is beneficial given that the Darby study showed a linear relationship between mean heart dose and the risk of a major cardiac event.^21^

One limitation of the script is in conjunction with the Beam Modulator MLC. Owing to the reduced field size of 16 cm by 21 cm, the collimator must be rotated through 90° in order to treat the entirety of the breast. The consequence of this is that the MLC leaves are deployed in the superior/inferior direction for step‐and‐shoot; at the moment, the Pinnacle^3^ block tool is incompatible with this. In practice, it is necessary for the planner to manually position the MLC leaves based on isodose volumes created by the script; segment weight optimization can then proceed. Useful planning time reductions are therefore not observed for the Beam Modulator MLC; the mean (range) reduction was measured to be 0.7 min (0.0–3.1 min).

Application of this partial automation technique to other sites is currently being investigated. A simpler version of the MLC segment creation method has been implemented and the optimization objectives adjusted in order to give PTV dose coverage. In the context of pelvis planning, the technique has been demonstrated to reduce step‐and‐shoot planning times by up to 15 min. For more complex sites for which class solutions with constrained gantry and collimator parameters are less likely, for example, abdomen or lung, challenges arise for the automation technique such as selecting the most appropriate fields for step‐and‐shoot purposes or automating the creation of dose sparing structures for use in the optimization.

It is inevitable that simple partial automation techniques will be superseded by more sophisticated commercial solutions. The method described by Purdie et al. and implemented in the RayStation treatment planning system is such an example.^13^ Nevertheless, as we show with this study, there is a role for noncomplex, noncommercial techniques to provide planning efficiencies until it becomes feasible for commercial approaches to be implemented. On a per plan basis, the benefit of such decreases is relatively modest. However, in the context of the yearly departmental workload for breast planning, this small benefit has the potential to translate into much greater planning efficiency.

## 5 Conclusion

The SPICE autosegmentation software offers a robust solution to automatic heart and lung delineation for breast patients and enables plan adjustment based on heart doses without introducing the burden of manual outlining. Partial automation of the breast radiotherapy treatment planning procedure through Pinnacle^3^ scripting facilitates reductions in planning times without compromising plan quality.
